# The spatial distribution of esophageal and gastric cancer in Caspian region of Iran: An ecological analysis of diet and socio-economic influences

**DOI:** 10.1186/1476-072X-10-13

**Published:** 2011-02-15

**Authors:** Mohammadreza Mohebbi, Rory Wolfe, Damien Jolley, Andrew B Forbes, Mahmood Mahmoodi, Robert C Burton

**Affiliations:** 1 Department of Epidemiology and Preventive Medicine, Faculty of Medicine, Nursing and Health Sciences, Monash University, Melbourne, Australia; 2 Department of Epidemiology and Biostatistics, School of Public Health, Tehran University of Medical Sciences, Tehran, Iran

## Abstract

Recent studies have suggested a systematic geographic pattern of esophageal cancer (EC) and gastric cancer (GC) incidence in the Caspian region of Iran. The aims of this study were to investigate the association between these cancers and the region's dietary and socioeconomic risk factors and to map EC and GC after adjustment for the risk factors and the removal of random and geographic variations from area specific age standardised incidence ratios (SIRs).

We obtained cancer data from the Babol cancer registry from 2001 to 2005, socioeconomic indices from the Statistical Centre of Iran, and dietary patterns from the control group in a case control study conducted in the study region. Regression models were fitted to identify significant covariates, and clusters of elevated rates were identified.

We found evidence of systematic clustering for EC and GC in men and women and both sexes combined. EC and GC SIRs were lower in urban areas, and were also lower in areas of high income. EC SIRs were lower in areas with higher proportions of people having unrestricted food choice and higher in areas with higher proportions of people with restricted food choice.

EC and GC were associated with aggregated risk factors, including income, urbanisation, and dietary patterns. These variables represent the influence of improved lifestyle which has coincided with a decrease in upper gastrointestinal cancer frequency over recent decades but which has not necessarily been uniform throughout the region.

## Introduction

Iran has high rates of both EC (esophageal cancer) and GC (gastric cancer) [1,2]. There is evidence of sharp gradients in incidence rates over relatively short geographical distances in the Caspian region of Iran [3]. While EC incidence has decreased to less than half the rate reported three decades ago [4], a recent study highlighted the existence of a strong systematic geographical pattern in EC and GC incidence in the southern region of the Caspian Sea, but did not consider area-related risk factors for analytical purposes [5]. In this study we investigate the association between the geographic pattern of EC and GC incidence and the dietary and socio-economic patterns in this region.

**Figure 1:**
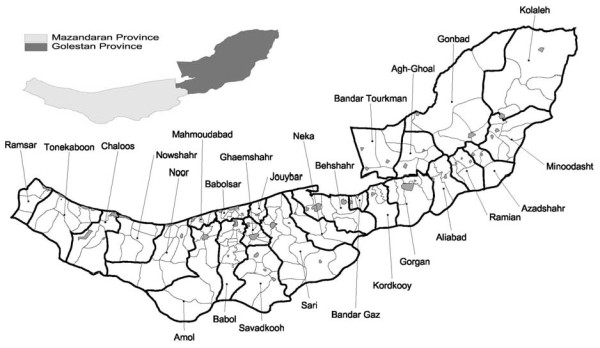
**Geographic boundaries of wards (bold polygons), and cities (gray polygons) and rural agglomerations within wards, in Mazandaran and Golestan provinces**.

The study region has a total population of 4.5 million (1.6 million in Golestan province, the reminder in Mazandaran province) [6]. The provinces of Iran are subdivided into wards. There are usually a few cities and rural agglomerations in each ward. Rural agglomerations are a collection of a number of villages. Currently, Mazandaran province has 15 wards, 46 cities and 110 agglomerations and Golestan province has 11 wards, 24 cities and 50 agglomerations. Figure 1 shows geographic boundaries of cities and rural agglomerations within wards in the two provinces.

A greater incidence of both EC and GC has been shown to occur in populations with low socio-economic status, SES [7]. This may be accounted for by the relationship between socioeconomic indicators and environmental exposures, occupational exposure and individual habits [8].

Observational studies have found that fruit and vegetable consumption generally protects against EC and GC risk, with stronger support for this association coming from case-control studies than from cohort studies, whereas salt, processed meats and foods, and sweets have usually been linked with increased risk of the disease [9-12]. Analysing dietary patterns can elicit a role of overall diet in EC and GC etiology, an association which has been demonstrated in previous studies [13-15].

This article reports the application of a five-part methodology as follows: (1) calculate and map sex-stratified age-standardised incidence ratios (SIRs) for EC and GC; (2) use appropriate statistical measures to evaluate geographic autocorrelation; (3) identify major socio-economic and dietary patterns in the study region; (4) evaluate the association of SES (socioeconomic status) and dietary patterns with EC and GC using multilevel modelling; and (5) compare maps of model adjusted smoothed estimates with the maps in part (1) that are not adjusted for geographic correlation or SES and dietary patterns.

## Methods

The study was ecological in design, and used census derived area data, map data, and individual person data as described below.

### Study Population

The estimated midyear population of Mazandaran and Golestan provinces between 2001 and 2005, stratified for sex, age in five-year intervals, and place of residence were obtained from the statistical centre of Iran [6]. These estimates were projections for 2001 to 2005, based on 1995 census data using the 2000 geographic boundaries [16,17]. Geographic coordinates for each agglomeration were also obtained that approximately reflected the geographical centroid of each agglomeration [6].

### Data sources

The cases of interest were all EC and GC patients registered between 2001 and 2005 among the study population. Data on incident cases of cancer were obtained from the Babol Cancer Registry; issues related to methods, quality and completeness of data collection for this cancer registry are described elsewhere [5,18]. In summary, the major sources of data collection related to cancer in the Babol cancer registry were reports from pathology laboratories, hospitals, and radiology clinics. Coding of cancer diagnosis samples was based on the international classification of disease for oncology (ICD-O) coding [19] and was done under direct supervision of pathology specialists. Microscopic verification was available for 47.7% of esophageal and 49.6% of gastric cancer cases. The reference address for all cases was the address at diagnosis. About 3% of cases lacked residential information at the agglomeration level. In order to use the cases with unknown residential information, the geographic referral pattern for each hospital or diagnosis centre was used to assign residences on a proportional as-likely basis. Concordance of residential place information within one year of diagnosis was examined for patients with multiple records during 1998-2000. Agreement on place of residence between the first diagnosis record and the next was 94% for gastric and 92% for esophageal cancer [20].

Explanatory variables were classified into two groups: Socio-economic characteristics of the 152 agglomerations and dietary patterns of the 26 wards. For each agglomeration the following socio-economic variables were obtained from the 1995 statistical yearbooks of Mazandaran and Golestan [16,17] or the income and expenses survey in urban and rural area in 1995 [21,22]: population density (inhabitants per square kilometre), relative level of activity (a synthetic indicator devised by the statistical centre of Iran that is calculated from the number of households, number of telephone lines, number of bank offices, number of commercial licences, electricity consumption, annual construction budget), annual income per family, annual expenditure on food per family, annual expenditure on fruit and vegetables per family, percentage of occupation in the industrial sector, percentage of occupation in the services sector, percentage of occupation in the agricultural sector, percentage of occupation in the construction sector, percentage of male unemployment, percentage of illiteracy. In addition to rural villages, some agglomerations contain one or more cities; a proportional as-likely basis method was used to calculate socio-economic characteristics of these agglomerations.

The dietary pattern of each ward was calculated using information on controls from a case control study [23,24]. Cases in that study were all esophageal, gastric and colorectal cancer patients registered by the Babol cancer registry from September 1993 to September 1996. Each case had one hospital and two neighbourhood based controls matched for age (within +/- 5 years) and sex. A structured food frequency questionnaire (FFQ), including 63 food and beverage items of interest, was used to evaluate dietary habits of the controls. The usual frequency of consumption of each food item was asked and answers were in terms of number of times per day, week, month, or year. We transformed the control data into average monthly intake for every food item, by assuming 1 month equal to 30 days. To reduce complexity we grouped the individual items into 18 separate food groups as shown in Table 1. Grouping was based on the similarity of nutrient profiles or their association with cancer. A total of 2322 (1154 female) controls had complete data available for the dietary pattern analysis (4.6% were excluded due to missing data). This sample was sufficient to provide good coverage of the study population at the ward level but not at the agglomeration level.

**Table 1:** Dietary pattern loadings from factor analysis (Restricted and Unrestricted food choice) of dietary consumption

	Rotated Component Matrix*	
**Items**	**Components**	
	**Unrestricted food choice**	**Restricted food choice**

Fresh and frozen fish	.848	-
Total fruit	.748	-.120
Sweets	-.261	.215
Poultry	.444	-
Red meat, liver	.230	.180
Salted/preserved food	-	.631
Potatoes: baked, boiled	-	.561
Canned fish	-	.516
Regular fibre	.112	-.254
Eggs	-	.279
White bread, rice, pasta	. 241	.653
Total vegetables	.427	-
Soft drinks	-	-
French fries	.183	-
Dairy	-	.212
Nuts	-	-.179
Pickles	-	.113

* Loadings less than 0.10 in absolute value are not displayed

### Factor analysis of socio-economic and dietary variables

A factor analysis was performed to summarise socio-economic information into a few uncorrelated factors. Factor analysis was also used for diet variables. Principal components followed by Varimax rotation with Kaiser normalisation was used to facilitate interpretation of the factors. The Anderson-Rubin method was used to create factor scores from the factor solution. The factors extracted with this method are uncorrelated with a zero average and variance of one [25]. We attached labels to the factors by considering the interpretation of items with sizable pattern coefficients. All factor scores were divided into sextiles for illustration purposes. Factor scores extracted from dietary patterns were divided into tertiles for all controls and the percentage of controls in each ward with factor scores in the highest tertile (3^rd^) was used in the regression model. For socio-economic components, factor scores related to each agglomeration were used in the regression model as a continuous covariate.

### Standardised incidence rates (SIR) calculation

Adjustment of incidence rates for differences in the age and sex structure of agglomerations was accomplished by sex-stratified age-standardisation (in 5-year intervals of age). The SIR for a certain agglomeration was obtained from the ratio of the observed and expected number of cases in that agglomeration. We used the indirect method of standardisation for internal comparisons [26]. Since the population of the region was stable between 2001 and 2005, the 2003 population size was used for computing the incidence rates in age and sex categories of the overall region and the subsequent expected number of cases in each agglomeration. In order to compare the incidence rates in the Mazandaran and Golestan region with other parts of the world, directly standardized incidence rates were also calculated, using the 1970 Segi's World population for historical comparisons [27], and 2000 WHO World Population for contemporary comparisons [28].

### Exploratory spatial data analysis

Two methods were used to measure spatial aggregation of the agglomeration SIRs; Moran's I [29] and semivariogram [30].

Moran's I is a correlation-type index based on continuous data values, but its interpretation is different from conventional correlation coefficients which take values in the range (-1, 1). The numeric scale of Moran's I is related to its expected value, E(I), under a random spatial pattern. Values less than E(I) are typically associated with a uniform/dispersed pattern and values greater than E(I) typically indicate a clustered pattern. We adjusted Moran's I for agglomeration counts by comparing the observed count in an agglomeration with its expected value under the constant risk hypothesis [31].

A graph of a semivariogram plotted against separation distance gives information about the geographical variability of the SIRs. If SIRs close together are more alike than those farther apart, a semivariogram plot increases as the separation distance (in kilometre) increases reflecting decreasing spatial autocorrelation. The height of the jump of the semivariogram at the discontinuity at the origin is called the nugget. Often, the semivariogram will level off to nearly a constant value (called the sill) at a large separation distance (called the range). Beyond this distance, observations are spatially uncorrelated. To obtain a succinct statistical description of the spatial correlation in the data we fitted three different parametric models (exponential, Gaussian, and spherical) to the empirical semivariogram, each of which can be described in terms of nugget, partial sill and range parameters [32]. The model we considered most appropriate was that which minimized the residual sum of squares between the theoretical model and the empirical semivariogram.

### Ecologic regression model incorporating spatial correlation

We assumed that, conditional on spatial random effects (u_ij_), the number of cancer cases in the I wards and J agglomerations within wards, Y_11_,... , Y_IJ _were independent Poisson random variables each with mean μ_ij_. A multilevel generalised linear model (MGLM) for the number of cases was specified as

(1) $$\log(\mu_{\text{ij}})=\log({\text{E}}_{\text{ij}})+\beta_{0}+{\text{X}}_{\text{ij}}{}^{\text{SES}}\beta_{\text{SES}}+{\text{X}}_{\text{j}}{}^{\text{diet}}\beta_{\text{diet}}+{\text{u}}_{\text{ij}}$$

where the offset term log(E_ij_) was the (log of the) expected number of cases for the j^th ^agglomeration in the i^th ^ward (assumed fixed), X^SES ^and X^diet ^were that agglomeration's rows from design matrices for the socio-economic and dietary factors, respectively; β_0 _was the intercept, and β_SES _and β_diet _were vectors of coefficients describing associations with the socio-economic and dietary factors, respectively [33]. Since SIR = μ_ij_/E_ij_, this is a model for agglomeration level SIRs with exp(β) interpretable as relative risk parameters within each agglomeration. Exploratory spatial data analysis showed evidence of both distance-based and neighbourhood-based geographical autocorrelation. To complete the model specification, we made distance-based and neighbourhood-based correlation structures for the spatial random effects u_ij_. We assumed that the vector of random effects followed the multivariate normal distribution MVN(0, Σ_u_(θ)), with the elements of Σ_u_(θ) defined as either conditional autoregressive (CAR) [34] or spatial point referenced (SPR) structures [35].

For the CAR-type model, we employed the intrinsic conditional autoregressive structure in which Σ_u_(θ) = ρW, with W being a spatial proximity matrix containing binary connectivity elements.

For the SPR-type model however, we assumed

(2) $$\Sigma_{\text{u}}(\theta )=\sigma^{2}\text{H}(\Phi )+\tau^{2}\text{I}$$

where H(.) is a correlation matrix depending on a parameter Φ. Exponential, spherical and Gaussian semivariogram models were used to describe the elements of Σ_u_(θ) as a function of nugget (τ^2^), partial sill (σ^2^), and range (Φ) parameters with the parametric form determined by empirical semivariogram analysis.

### Model comparison

The -2 Log-Likelihood and two most commonly used penalized model selection criteria, the Bayesian information criterion (BIC) and Akaike's information criterion (AIC), were used for model comparison.

### Cartographic display

**Figure 2:**
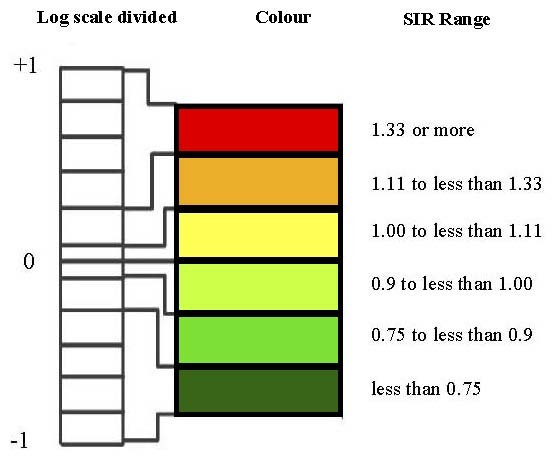
**Standardised incidence rate (SIR) categories**.

In this study the RR (risk ratio) break points were determined by considering values in the range 0.1 to 10. This corresponds to the range -1 to +1 upon logarithmic transformation. Then this logarithmic scale was divided into 11 equal intervals centred on zero, the break point values were transformed back to the original RR scale, and the five middle intervals were used in the maps. As shown in ​Figure 2, the middle category was further divided above and below 1. A red-green colour scheme was used for the maps, with shading of red for areas with the highest SIR (>1.33), followed by orange and yellow for areas with moderately elevated SIR, light and medium green for areas with moderately low SIR, and dark green representing areas with the lowest SIR (<0.75).

### Software

SIR calculation was performed in Microsoft Excel, exploratory spatial analyses were performed using SAS's VARIOGRAM Procedure [36], factor analyses were conducted in SPSS 17 and the SAS Glimmix procedure was used to carry out MGLM regression [37,38].

## Results

### Factor analysis

Dietary factors: Table 1 shows factor loadings of the 17 food group items on the two factors with eigenvalues greater than 0.1. The first dietary pattern, accounting for 13% of the variability, was characterized by high intake of foods generally thought to be preventive including vegetables, fruit, fish, and regular fibre, and was thus labelled "unrestricted food choice diet," whereas the second dietary pattern, accounting for 8% of the variability and labelled "restricted food choice diet," was characterized by high consumption of processed/salted meat, sweets, potatoes, soft drinks and low consumption of fish, fruit and vegetables.

Socio-economic factors: Factor analysis identified three factors with eigenvalues greater than 0.1. Table 2 shows the correlations between socio-economic items and the extracted factors. The three factors account for 53% of total variance in socio-economic variables and individually the factors account for: income: 25%, urbanisation: 15% and literacy: 13%.

**Table 2:** Socio-economic loadings from factor analysis (Income, Urbanisation and Literacy)*

		Rotated Component Matrix	
**Items**		**Components**	
	**Income**	**Urbanisation**	**Literacy**

Annual income per family	.846	-	-
Annual expenditure on food per family	.654	.165	-
Annual expenditure on fruit and vegetables per family	.455	.151	-
Population density	-	.285	-
Relative level of activity	.318	.221	.533
% of male unemployment	-.321	-.679	-
% of employment in agriculture	-.213	-.808	-
% of employment in industry	.199	.341	-
% of employment in construction	-.208	-	.470
% of employment in services	.189	.824	-.198
Female illiteracy	-	-	-.642
Male illiteracy	-	-	-.669

*Loadings less than 0.10 in absolute value are not displayed

### Exploratory analysis

**Figure 3:**
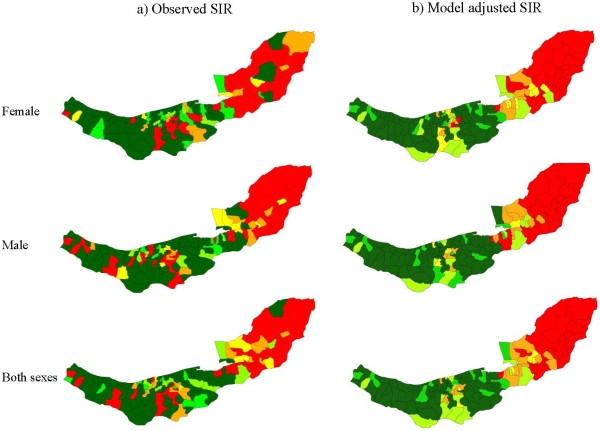
**Observed spatial pattern (a), and model adjusted spatial pattern (b) of esophageal cancer's SIR in female, male and both sexes combined**.

**Figure 4:**
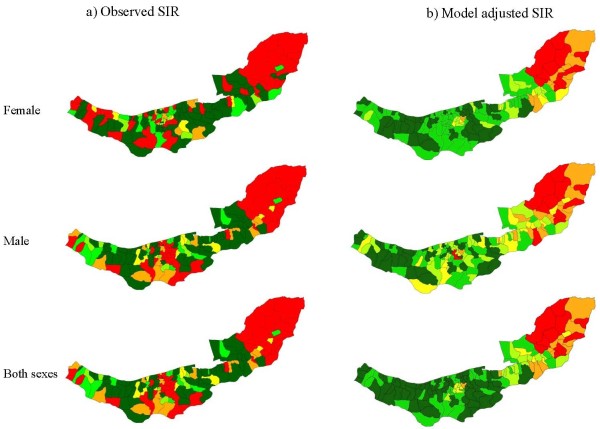
**Observed spatial pattern (a), and model adjusted spatial pattern (b) of gastric cancer's SIR in female, male and both sexes combined**.

A total of 5826 new gastrointestinal cancer cases were diagnosed in 2001-2005 in Mazandaran and Golestan. Of these, 1693 cases were diagnosed with EC and 2665 were GC. Table 3 shows incidence rates, number of cases and Moran autocorrelation indices by site of the cancer and sex. For both cancer sites the observed Moran indices were greater than their expected values, which indicated systematic cluster patterns for EC and GC in the region. Consistent with Moran's I, Figures 3(a) and 4(a) showed strong spatial aggregations in EC and GC for males, females and both sexes combined, with a tendency for high rates in the eastern and central agglomerations and low rates in the west.

**Table 3:** Incidence rate, directly standardized incidence rates (per 100,000 person-years using the 1970 and 2000 world population) and Moran's I autocorrelation for esophageal and gastric cancers in Mazandaran and Golestan provinces of Iran

Cancer Type	Sex	No. of Cases	Incidence Rate	1970 world population	2000 world population	Moran's I*
	Male	891	8.10	12.16	14.61	0.28
Esophageal	Female	810	7.23	11.27	12.73	0.30
	Both sexes	1693	7.67	11.72	13.71	0.22
	Male	1838	15.62	23.04	26.78	0.22
Gastric	Female	827	6.46	9.92	11.25	0.12
	Both sexes	2665	11.04	16.50	19.02	0.26

* E(I) for all tests are -0.0066, and p-values for Moran's I were less than 0.001 for all analyses

**Figure 5:**
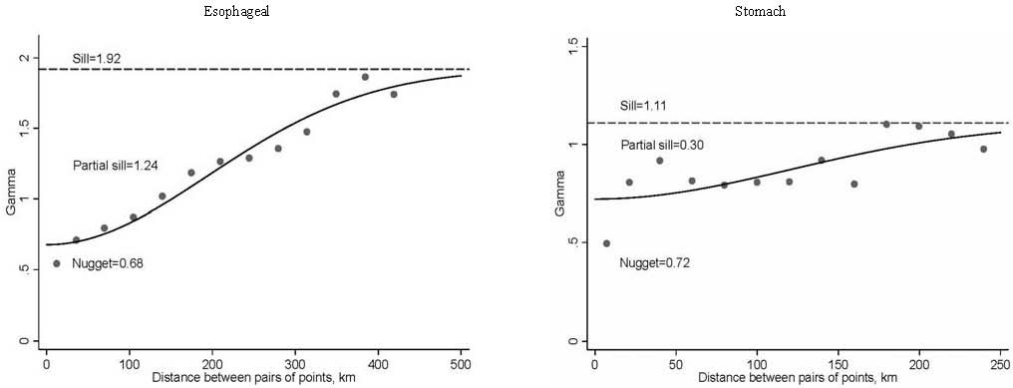
**Gaussian semivariograms fit to the residuals-based empirical semivariograms for both sexes combined in esophageal and gastric cancer**.

A Gaussian semivariogram best fitted the empirical semivariogram for both cancer sites as illustrated in Figure 5 for both sexes combined (the findings were similar for males and females separately). We found that the effective range of spatial autocorrelation for EC was 360 km, which was shorter than the range of spatial autocorrelation for GC (428 km). The nugget/sill ratios were 0.35 and 0.65 for EC and GC respectively, indicating moderate degrees of spatial autocorrelation. In addition, no major trends of mean and variance were observed with direction in either cancer site; therefore, the semivariogram between any two locations depended only on the distance between them, not their exact locations.

### Ecologic regression

Comparison between nonspatial and spatial regression approaches is provided in Table 4. The comparison, which was based on the likelihood ratio, AIC and BIC, indicated that in general the conditional autoregressive autocorrelation structure had a better fit to observed data than the competing Poisson regression models.

**Table 4:** Comparison of model goodness of fit using nonspatial Poisson regression and spatial Poisson models with conditional autoregressive (CAR), and spatial point referenced (SPR), autocorrelation structures

	Model		-2Log-Likelihood	AIC	BIC
Esophageal cancer	Poisson regression with uncorrelated random effect	Female	511.3	517.2	521.8
		Male	498.2	501.3	510.9
		Both sexes	453.2	455.5	458.4
		
	Spatial Poisson regression with Gaussian SPR correlation function	Female	481.3	485.2	491.6
		Male	453.7	455.0	460.3
		Both sexes	446.5	448.5	451.5
		
	Spatial Poisson regression with CAR correlation function	Female	470.9	476.9	485.9
		Male	411.5	417.5	426.5
		Both sexes	332.0	338.0	347.0

Gastric cancer	Poisson regression with uncorrelated random effect	Female	560.5	566.5	575.5
		Male	485.2	488.0	491.2
		Both sexes	463.4	468.3	471.8
		
	Spatial Poisson regression with Gaussian SPR correlation function	Female	483.2	488.0	491.1
		Male	469.1	471.0	580.5
		Both sexes	447.8	453.8	462.8
		
	Spatial Poisson regression with CAR correlation function	Female	511.3	519.6	523.4
		Male	467.0	473.0	482.1
		Both sexes	384.0	386.0	389.0

**Figure 6:**
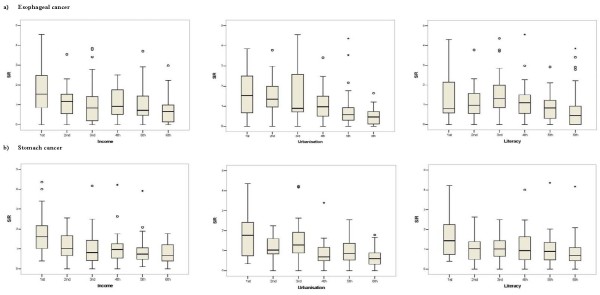
**Relationship between esophageal (a) and gastric (b) cancer SIRs, and sextiles of the following three socio-economic score factors: income, urbanisation, and literacy**. Each boxplot within each panel displays the distribution of the SIRs within that sextile.

**Figure 7:**
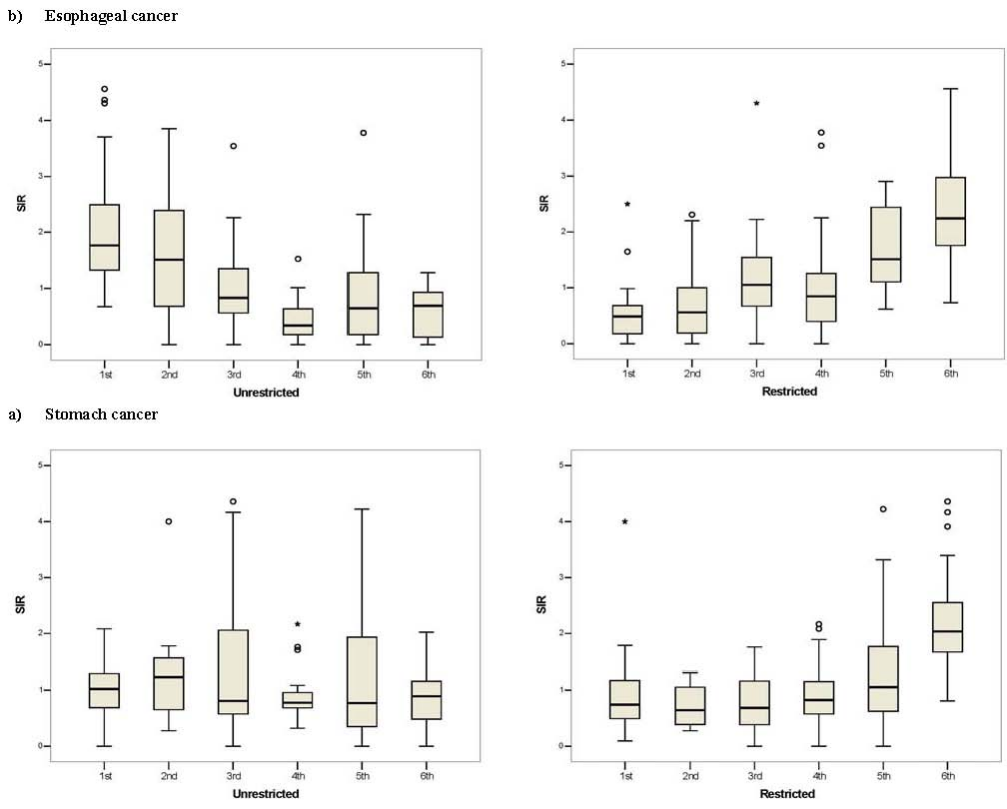
**Relationship between esophageal (a) and gastric (b) cancer SIRs, and sextiles of the following two dietary pattern score factors: unrestricted food choice, and restricted food choice**. Each boxplot within each panel displays the distribution of the SIRs within that sextile.

Figures 6 and 7 display boxplots of the SIRs by sextile of socio-economic and dietary factor scores. Overall these figures suggest moderate dose-response associations between the socio-economic and dietary factor scores and EC and GC. Confirmation of these associations comes from the results for the multilevel Poisson models for male, female and both sexes in Table 5. For men and women combined, increasing EC SIR in an agglomeration was associated with: decreasing percentage of ward-specific population in the 3^rd ^study-area tertile of the unrestricted food choice factor, increasing percentage in the 3^rd ^tertile of the restricted food choice factor, and decreasing scores of the income and urbanisation factors (p < 0.001). Increasing SIR in an agglomeration for GC was associated with decreasing income score for men and women separately, and urbanisation factors, for men and women combined, (p < 0.001). In addition, for GC the analysis of both sexes combined showed weak associations with the percentage in the 3^rd ^tertile of the unrestricted food choice factor, the percentage in the 3^rd ^tertile of the restricted food choice factor, and the income and literacy factors (p-values in range 0.05 to 0.1). Model smoothed SIR maps after adjustment for covariates from Table 5 with p-value less than 0.1 are illustrated in Figures 3(b) and 4(b).

**Table 5:** Parameter estimation for SES and dietary patterns

			Esophageal cancer				Gastric cancer		
			
	Factor	RR	95% CI		P-value	RR	95% CI		P-value
							
			lower	upper			lower	upper	
Female	Unrestricted food choice*	0.91	0.84	0.99	0.04	0.89	0.67	1.18	0.42
	Restricted food choice*	1.27	1.13	1.44	<0.001	1.08	0.87	1.34	0.49
	Income	0.78	0.68	0.90	<0.001	0.66	0.56	0.77	<0.001
	Urbanisation	0.71	0.62	0.81	<0.001	0.86	0.76	0.97	0.02
	Literacy	0.94	0.84	1.06	0.25	0.98	0.86	1.11	0.83
	
Male	Unrestricted food choice*	0.75	0.68	0.82	<0.001	0.91	0.64	1.31	0.31
	Restricted food choice*	1.15	1.05	1.26	0.004	1.09	0.91	1.31	0.33
	Income	0.85	0.78	0.93	0.009	0.75	0.65	0.87	<0.01
	Urbanisation	0.76	0.69	0.83	<0.001	0.90	0.75	0. 91	0.02
	Literacy	0.94	0.79	1.13	0.84	0.95	0.78	1.16	0.30
	
Both sexes	Unrestricted food choice*	0.81	0.75	0.88	<0.001	0.92	0.84	1.00	0.05
	Restricted food choice*	1.36	1.24	1.49	<0.001	1.08	0.97	1.20	0.09
	Income	0.86	0.80	0.93	<0.001	0.92	0.83	1.03	0.06
	Urbanisation	0.83	0.77	0.89	<0.001	0.73	0.68	0.84	<0.001
	Literacy	0.96	0.89	1.04	0.32	0.88	0.76	1.01	0.08

* Proportion of individuals in each ward with factor scores in the highest tertile

## Discussion

In this ecologic study we observed statistically significant associations between agglomeration-specific EC and GC SIR and SES and dietary patterns. We hypothesised that strong geographical EC and GC risk patterns highlighted in previous studies [3,5] could be explained by the existence of important geographical differences in the prevalence of two well-established and modifiable risk factors (SES and dietary pattern).

Two dietary patterns were identified: "restricted food choice" and "unrestricted food choice" that explained approximately 21% percent of the variance in responses to the FFQ. The unrestricted food choice pattern was positively correlated with total fruit, total vegetables, seafood, poultry and regular fibre, and negatively correlated with sweets. This dietary pattern was linked to an inverse risk of EC in male, female and both sexes combined. The restricted food choice was negatively correlated with total fruit and regular fibre, positively correlated with salted and preserved foods and had very small factor loading on total vegetables, seafood and poultry. This dietary pattern was associated with higher risk of EC in male, female and both sexes combined; Low intake of fruit and vegetables has been consistently associated with higher risk of EC with a meta-analysis suggesting that protective effects were more pronounced for fruit than vegetables [9]. Families in the regions of high incidence of EC in our study reported very limited intake of fruit and vegetables relative to families in the low incidence areas, consistent with a case-control study in the region that showed a higher intake of raw vegetables reduced the risk of esophageal cancer by 40-50% [39].

The restricted food choice was linked with GC increase in both sexes combined. We also found a high intake of salted/preserved meat, canned fish and pickles was associated with increased GC risk in both sexes combined.

A link between certain demographic and economic features of regions and the risk for EC and GC has been shown in several studies [7,8]. The socio-economic variables used in our study enabled three such indices to be studied: income, urbanisation and literacy. We found higher incidences of EC and GC in men and/or women were related to lower annual income, lower annual expenditure on food, lower annual expenditure on fruit and vegetables, higher percentage of unemployment, and higher percentage of employment in agriculture and construction sectors. Both cancer sites analysed in this study had higher SIR in the rural setting. This association may be related to lower SES, higher unemployment and high levels of farming in rural agglomerations.

In our study, expenditure on food in general and expenditure on fruit and vegetables had large positive factor loadings on the income and urbanisation indices. In addition, income and urbanisation indices were positively correlated with unrestricted food pattern and negatively correlated with restricted food pattern. This correlation was stronger in the eastern region, especially in the Turkmen plain. Therefore, lower SES was linked to a diet deficient in fruit and vegetables in rural agglomerations, which is an important risk factor for EC and GC. An increased risk of gastric cancer associated with agricultural occupations has been consistently reported, and exposure to pesticides, organic and inorganic dusts, fertilizers, and nitrates has been suggested as the major contributing risk factors [40-42]. There is no Pesticide Register in Iran to compile information on the use of these products. As a result, specific ecological indicators cannot be used to measure the populations' exposure to pesticides. Consequently, the percentage in agricultural occupations, where pesticide exposure could be assumed to be higher, and the urbanisation score were used as indirect indicators of the use of pesticides in agglomerations. We found a significant negative association between EC and GC risk and urbanisation score.

Some details of our study methods require discussion. First, the exact timing of SES and diet-related exposures and cancer occurrence is important for our study. The lag time between risk factors exposure and EC and GC cancer development was ascertained for 3 large prospective cohort studies involving more than half a million men and women [43-45]. In these prospective cohort studies a lag time between 6 to 12 years was long enough for the development of EC and GC in healthy participants, and, more importantly, to find a significant association between SES and dietary exposures and EC and GC cancer occurrence. Our study had an average lag time of 10 years, with a range of 6-12 years, between exposure measurements (1993-1996) and outcomes (2001-2005), which is consistent with these findings.

Second, could human migration in the study region have caused enough selection bias to influence the result? It is known that external migrants to the study region have lower incidence of EC and similar GC incidence to the national rate [46]. Between the 1995 and 2005 censuses 556,455 people (on average 1.4% per annum of the study population) migrated to the study region. Most immigrants (83%) were healthy labour force participants and their younger relatives, explaining the lower cancer rates of migrants. However, external migration from other provinces, occurring mainly to the major cities of the study region, was accountable for only 29% of total migration with internal migration accounting for the reminder. It seems unlikely that these modest migration figures would strongly influence the observed associations.

Third, controls from a local case-control study were used to identify dietary patterns. The number of controls per wards ranged from 26 in the low populated ward Bandar Gaz to >250 for wards with major cities like Babol [24]. In order to find any selection bias due to percentage of coverage in different wards or urban and rural areas we compared age, residential place (urban/rural), sex and ward distribution of cases with EC and GC incidence for 2003 to 2006 period. There was no significant difference in these demographic characteristics between controls from the case control study and cases on the registry. About one third of the controls were selected as neighbouring the cases in the case-control study. This mechanism of control selection possibly obtained a non-random representation of dietary habits in wards. This may the dilute association between EC and GC and dietary patterns.

Fourth, in this study SES and dietary pattern scores were used as markers of the heterogeneous distribution of lifestyle and dietary factors influencing EC and GC risk. Selection of these variables was limited by the availability of information at agglomeration or ward level, so they only partially reflect the distribution of related risk factors. However, their inclusion served to smooth SIR, taking into account both the spatial relation among agglomerations and the variability associated with these indices.

Fifth, justification of sample size is necessary. For factor analysis it is recommended that five subjects per item, with a minimum of 100 subjects regardless of the number of items is a sufficient sample size [47]. There were 17 food items and 2322 subjects in the dietary pattern analysis and 12 Socio-economic items and 152 units (agglomerations) for the SES factor analysis, and so these met the minimum sample size criteria. To the best of our knowledge no study has focused on sample size and robustness issues in multilevel Poisson regression in a comprehensive manner. However, results from a simulation study suggest that for generalised linear mixed models with low prevalent events at least a minimum of 100 groups and 30 to 50 individuals per group were necessary [48]. Our study contained 152 groups (agglomerations) and a mean of 11 and 16 cases for EC and GC. While the group size was large enough for accurate regression parameter estimation, small sample size within agglomerations suggested possible bias in the second level standard errors.

Ecologic studies are perhaps best considered to be hypothesis generating, although small area analysis tends to reduce ecological fallacy, since the populations defined by agglomerations boundaries are more homogeneous. While this might well be true of villages and towns of average size, in large cities this may not be so. However, the results reported here correspond to an overall mean, and socio-economic and dietary patterns differences inside cities have been disregarded. It would be interesting to extend our work by assessing whether such differences exist in major cities, such as Sari, Ghaemshahr and Gorgan.

## Conclusion

Multilevel spatial modelling revealed associations between EC and GC incidence and SES and dietary indices. High EC and GC incidence and low SES scores often coincided in rural areas. Higher prevalence of restricted food choice was associated with higher EC in the eastern agglomerations, especially in the Turkmen plain. Our study revealed that there were systematic geographical variations in EC and GC SIRs across the Caspian region, and particularly an elevated risk in contiguous high-risk eastern areas. Further studies targeted to specific regions could help to identify the risk factors that may contribute to the geographical patterns in EC and GC SIR's identified here.

## Abbreviations used

AIC: Akaike's information criterion; BIC: Bayesian information criterion; CAR: conditional autoregressive; EC: esophageal cancer; FFQ: frequency questionnaire GC: gastric cancer; MGLM: multilevel generalised linear model RR: risk ratio; SES: socioeconomic status; SIR: standardised incidence ratio; SPR: spatial point referenced

## Competing interests

The authors declare that they do not have competing interests.

## Authors' contributions

MMo and RW designed and conducted the study. MMo was responsible for the data collection process and issues related to data quality. DJ, AF, MMa and RB assisted in designing and conducting the study. MMo performed the statistical analysis. MMo and RW wrote the first draft of the manuscript to which all authors subsequently contributed. All authors read and revised the manuscript for important intellectual content and approved the final manuscript.
